# The Inheritance of the Pheromone Sensory System in Two *Helicoverpa* Species: Dominance of *H. armigera* and Possible Introgression from *H. assulta*

**DOI:** 10.3389/fncel.2016.00302

**Published:** 2017-01-10

**Authors:** Meng Xu, Jun-Feng Dong, Han Wu, Xin-Cheng Zhao, Ling-Qiao Huang, Chen-Zhu Wang

**Affiliations:** ^1^State Key Laboratory of Integrated Management of Pest Insects and Rodents, Institute of Zoology, Chinese Academy of SciencesBeijing, China; ^2^College of Life Sciences, University of Chinese Academy of SciencesBeijing, China; ^3^College of Forestry, Henan University of Science and TechnologyLuoyang, China; ^4^Department of Entomology, College of Plant Protection, Henan Agricultural UniversityZhengzhou, China

**Keywords:** *Helicoverpa armigera*, *Helicoverpa assulta*, intraspecific hybridization, sex pheromone, olfactory sensory neurons, antennal lobes

## Abstract

Hybridization of sympatric closely related species may sometimes lead to introgression and speciation. The sister species *Helicoverpa armigera* and *Helicoverpa assulta* both use (Z)-11-hexadecenal and (Z)-9-hexadecenal as sex pheromone components but in reversed ratios. Female *H. armigera* and male *H. assulta* could hybridize and produce fertile male hybrids, which can then backcross with females of the two parent species to get backcross lines in the laboratory. In this study, we compared the olfactory responses to pheromone compounds in the periphery and in the antennal lobes (ALs) of males of the two species, as well as of their hybrids and backcrosses. Single-sensillum recordings were carried out to explore characteristics of male-specific sensilla on the antennae, and *in vivo* calcium imaging combined with digital 3D-reconstruction was used to describe what happens in the macroglomerular complex (MGC) of the AL. The results show that the population ratio of the two male-specific types of olfactory sensory neurons responding to two sex pheromone components are controlled by a major gene, and that the allele of *H. armigera* is dominant. Consistently, the study of the representative areas activated by sex pheromone components in the ALs further support the dominance of *H. armigera*. However, the topological structure of the MGC in the hybrid was similar but not identical to that in *H. armigera*. All subtypes of male-specific sensilla identified in the two species were found in the male hybrids and backcrosses. Moreover, two new subtypes with broader response spectra (the expanded A subtype and the expanded C subtype) emerged in the hybrids. Based on the inheritance pattern of the pheromone sensory system, we predict that when hybridization of female *H. armigera* and male *H. assulta* occurs in the field, male hybrids would readily backcross with female *H. armigera*, and introgression might occur from *H. assulta* into *H. armigera* through repeated backcrossing.

## Introduction

Hybridization is defined as interspecific mating and produces viable offspring. It has been reported to occur in many species of both plants and animals (Mallet, 2005; Baack and Rieseberg, 2007). Reproductive isolation between hybrids and parental species might be complete: hybrids escape from the homogenizing effects of gene flow from parental species, which may lead to the emergence of new species (Dowling and Secor, 1997; Rieseberg, 1997; Buerkle et al., 2000). On the other hand, when reproductive isolation does not take place because of lack of ecological and spatial isolation, infertile hybrids, or other reasons (McCarthy et al., 1995; Buerkle et al., 2000), then hybrids might backcross with their parents. Such a gene flow (introgression) through repeated backcrossing could be an important evolutionary drive (Baack and Rieseberg, 2007; Mallet, 2007). Interspecies mating and generation of hybrids often occur in related insect species, but the fate of hybrids has been largely ignored (Hansson et al., 1987; Roelofs et al., 1987; Baker et al., 2006; Vickers, 2006a,b; Domingue et al., 2008).

In closely related moth species, the sex pheromone olfactory system plays an important role in behavioral isolation due to its exquisite sensitivity and narrowly tuned responses. Male moths take advantage of highly specialized olfactory sensory neurons (OSNs) housed in antennal sensilla to detect female sex pheromones. The pheromonal information received by OSNs is then conveyed to the primary brain centers, the antennal lobes (ALs), which contain specialized glomeruli for processing sex pheromone information. The sex pheromone sensory system of Heliothine moths in the family Noctuidae are one of the best studied among Lepidoptera (Berg et al., 1995; Christensen et al., 1995; Hansson et al., 1995; Baker et al., 2004). However, little work has been done on the genetic mechanisms of pheromone detection. Experimental hybridization studies make it possible to identify the genetic changes responsible for differences between species (Dowling and Secor, 1997).

The genetic basis of the pheromone perception system in the European corn borer (*Ostrinia nubilalis*) is another thoroughly investigated arrangement (Hansson et al., 1987; Roelofs et al., 1987; Domingue et al., 2008). Electrophysiological recordings from the OSNs in hybrid males of two strains, E and Z, of *O. nubilalis* revealed that spike amplitude ratios of sex pheromone OSNs exhibit E-strain dominance and are controlled, at least partially, by sex-linked genes (Olsson et al., 2010). Further studies showed that the volume ratios of glomeruli in the macroglomerular complex (MGC) are sex-linked and co-dominant in the two strains (Karpati et al., 2010). However, the neural and behavioral responses showed no genetic linkage with sex pheromone biosynthesis (Roelofs et al., 1987; Lofstedt et al., 1989). Another well studied system is the two closely related species of Heliothine moths, *Heliothis virescens* and *Heliothis subflexa*. Response profiles of pheromone-sensitive OSNs of their hybrids ranged from being closely similar to those of parental types to being intermediate (Baker et al., 2006), while the behavioral phenotype of hybrid males linked to underlying central olfactory characteristics in the ALs was influenced by genetic factors inherited from both parental species (Vickers, 2006a,b).

*Helicoverpa armigera* and *Helicoverpa assulta* are two other sympatric sister species belonging to the heliothine sub-family. They use the same compounds, (Z)-11-hexadecenal (Z11-16:Ald) and (Z)-9-hexadecenal (Z9-16:Ald) as their main sex pheromone constituents but in almost reversed ratios, 98:2 and 5:95, respectively (Kehat et al., 1980; Cork et al., 1992; Wang et al., 2005). Accordingly, the population ratios of the OSNs of A, C type sensilla, responsive to Z11-16:Ald and Z9-16:Ald, respectively, are reversed in the two species (Berg and Mustaparta, 1995; Wu et al., 2013; Berg et al., 2014; Xu et al., 2016). In *H. assulta*, the C type sensilla species house two responding neurons. One large amplitude neuron responding to Z9-16:Ald, and one with small amplitude responding to Z9-14:Ald, and have been previously identified on the basis of their different spike amplitudes (Berg and Mustaparta, 1995; Berg et al., 2005). In *H. armigera*, the two neurons in C type sensilla are difficult to differentiate due to their similar spike amplitudes (Wu et al., 2015; Xu et al., 2016). As in other Heliothine species (Christensen et al., 1991; Berg et al., 1998; Vickers and Christensen, 2003), the pheromonal information received by OSNs is conveyed to the MGC area of the AL (Berg et al., 2005; Zhao and Berg, 2010; Wu et al., 2013, 2015). In *H. armigera*, the major sex pheromone component Z11-16:Ald elicits a response in the larger glomerulus of the MGC, the cumulus, while the minor component Z9-16:Ald activates one smaller glomerulus of MGC, the posterior dorsomedial unit (Dm-p) (Wu et al., 2013). Conversely, in *H. assulta*, the cumulus of the AL receives inputs from OSNs tuned to Z9-16:Ald, while the smaller glomerulus, ventral unit, is connected to OSNs tuned to Z11-16:Ald (Berg et al., 2005; Wu et al., 2013).

Besides Z11-16:Ald and Z9-16:Ald, some other compounds identified in female sex pheromone glands also play roles in pre-mating reproductive isolation between these two species (Xu et al., 2016). These interspecific signals are all detected by C type sensilla (Berg et al., 2005; Chang et al., 2016; Xu et al., 2016). Although all of such sensilla respond to Z9-16:Ald and Z9-14:Ald, their response profiles in *H. armigera* and *H. assulta* still present some differences. C type sensilla in *H. armigera* display additional responses to (Z)-11-hexadecenyl acetate (Z11-16:Ac) and (Z)-11-hexadecenol (Z11-16:OH) (Chang et al., 2016; Xu et al., 2016), while those in *H. assulta* are reported to be activated also by (Z)-9-hexadecenol (Z9-16:OH) and Z11-16:OH, plus (Z)-9-hexadecenyl acetate (Z9-16:Ac) (Berg and Mustaparta, 1995) and Z11-16:Ac (Xu et al., 2016). These differences and the availability of hybrids of the two species provides an ideal system to study the genetic basis of their sex pheromone communication (Wang and Dong, 2001; Wang et al., 2004; Tang et al., 2005, 2006).

*Helicoverpa armigera* and *H. assulta* can be successfully hybridized in the laboratory (Wang and Dong, 2001; Tang et al., 2005; Zhao et al., 2006). The cross between female *H. armigera* and male *H. assulta* yields F1 offsprings (RS hybrids), of which half includes fertile offsprings and half of malformed offsprings, with all the fertile offsprings being males. The malformed individuals were found lacking a penis, valva, or ostium bursae. The reciprocal cross between female *H. assulta* and male *H. armigera*, on the other hand, yielded both male and female fertile offsprings (SR hybrid). RS hybrids are much easier to produce than SR hybrids. Fertile hybrids can backcross with both parental species, but much more easily with *H. armigera* (unpublished data). The ratio of two principal pheromone components of both species, Z11-16:Ald and Z9-16:Ald, in the SR hybrid female glands is 100:4, which is very similar to that of *H. armigera* (Wang et al., 2005). Both behavioral and electroantennogram responses of hybrid males and backcross males to pheromone blends of these two species have also been studied previously (Zhao et al., 2006). *H. armigera* genes appear dominant in determining the behavioral responses. Such responses of backcrosses of male RS hybrids with two parental species were close to that of the backcross parents, while backcross of female SR hybrids showed reduced behavioral responses (Zhao et al., 2006). However, the OSN responses in A and C type sensilla in males of hybrids and backcross lines to sex pheromones are still unknown. Because male RS hybrids are much easier to produce in interspecific crosses, and that they also readily backcross with *H. armigera*, we hypothesize that introgression would direct to *H. armigera*, which will gain alleles from *H. assulta* by hybridization.

To understand the genetic basis of olfactory sensory system in these two species and characterize the gene flows during the hybridization and the fate of hybrids, we first carried out hybridization of *H. armigera* and *H. assulta*, and got hybrids and backcross lines. We then used males of these two species, plus hybrids and backcrosses to investigate the electrophysiological responses of OSNs in male-specific sensilla to sex pheromone components and related chemicals using single sensillum recordings. We finally studied the structure and response patterns of the MGC by calcium imaging measurement and digital 3D-reconstruction.

## Materials and Methods

### Hybrids and Backcrosses of *H. armigera* and *H. assulta*

*Helicoverpa armigera* and *H. assulta* were originally collected as larvae in tobacco fields in Zhengzhou, Henan province of China, and reared in the laboratory under a 16L: 8D photoperiod cycle at 26 ± 1°C and 55–65% relative humidity. Since these two species are agricultural pests in China, no specific permission was required to collect samples and carry out experiments. The larvae were fed on two kinds of artificial diets whose main component is wheat germ. Pupae were sexed and males and females were put into separate cages for eclosion. After eclosion, 50 individual couples of female *H. armigera* and male *H. assulta* and 50 couples of male *H. armigera* and female *H. assulta* were placed in cylindrical cages (diameter 28 cm, height 35 cm) to get the F1 hybrids (RS and SR). We performed 30 such experiments, but only got RS hybrids. Backcross offsprings, BC1 and BC2 were obtained by F1 (RS) males crossed with female *H. assulta* and *H. armigera*, respectively. Two- to five-day-old males were used for all the experiments.

### Chemicals

Ninety-two percentage of Z11-16:Ald, 90% Z9-16:Ald, 93% Z9-14:Ald, 92% Z9-16:OH, 92% Z11-16:OH, 90% Z9-16:Ac, and 92% Z11-16:Ac were purchased from Shin-Etsu Company (Tokyo, Japan). The purity of these compounds was increased to >98% by chromatography on a silica gel column. Solutions were prepared in HPLC-grade paraffin oil (Analytical grade, Fluka). All the solutions were stored in 2 mL glass vials (Agilent Technologies, Santa Clara, CA, USA) at -20°C.

### Single Sensillum Recording

The insect was placed inside a 1 mL disposable Eppendorf pipette tip with the narrow end cut to allow the head and the antenna to protrude. The head and antenna were immobilized with dental wax under a stereomicroscope and the reference electrode was inserted into a compound eye. Male-specific trichoid sensilla (Supplementary Figure S1), which have been shown to house pheromone-sensitive neurons in many heliothine moths (Berg and Mustaparta, 1995; Baker et al., 2004), were randomly selected from the 20th to 60th flagellar segment of the antenna, and their tips were cut off using custom sharpened forceps, and inserted into glass capillaries filled with receptor lymph saline (Kaissling and Thorson, 1980). An Ag-AgCl electrode was placed in the glass micropipette to record action potentials of the receptor neurons. The recorded signals were then amplified through an IDAC interface amplifier (IDAC-4, Syntech, Germany). The software Autospike, version 3.4 (Syntech, Germany), was used to store and analyze data.

A continuous stream of purified and humidified air was directed on the antenna (12.5 mL/s) from the outlet of a steel tube (i.d. 6 mm, length 15 cm), positioned 2 cm from the antenna. Test odors were injected into the air stream using a stimulus flow controller (CS-55, Syntech, Germany), which generated 200 ms air pulses through the odor cartridge at a flow rate of 10 mL/s, and a compensating air flow was provided to keep a constant current. Each odor cartridge contained a filter paper strip (0.7 cm × 2.5 cm) loaded with 100 μg (10 μL of 10 μg/μL solutions) stimulus and inserted in a 15 cm Pasteur pipette.

Firstly, the pheromone components Z11-16:Ald, Z9-16:Ald, and Z9-14:Ald were used to identify types A, B, and C sensilla. According to previous results (Xu et al., 2016), sensilla responsive to Z11-16:Ald are classified as A type sensilla, those responsive to Z9-14:Ald but not to Z9-16:Ald are B type and those responsive to both Z9-16:Ald and Z9-14:Ald are C type. Four additional compounds, Z9-16:OH, Z11-16:OH, Z9-16:Ac, and Z11-16:Ac were also used to collect responses. The spike frequencies (spikes/s) were calculated by counting the number of spikes with the same amplitude during the first 200 ms of the response. However, we counted the total number of spikes from the C type sensilla of *H. armigera* because our previous cross-adaptation test indicated this type sensilla of *H. armigera* contain two OSNs with similar spike amplitudes which are difficult to differentiate (Wu et al., 2015). For both non-responding and poorly responding OSNs, spikes were counted during the same 200 ms post-stimulus interval. For each individual, we recorded from at least 10 sensilla of A or C type.

### Calcium Imaging

Calcium imaging was adopted from Galizia and Vetter (2005). Preparation of moths and optical recording were performed as described previously (Wu et al., 2013). Briefly, virgin male moths were restrained in plastic tubes and fixed with dental wax. After exposing the brain, a calcium-sensitive dye, CaGR-1-AM (Molecular Probes, Eugene, OR, USA) was bath applied to stain the ALs. The dye was firstly dissolved in 20% Pluronic-127 in dimethyl sulfoxide and then diluted in Ringer solution to a final concentration of 30 μmol/L. The insect was then placed in the dark for 1 h at 12°C, and then thoroughly rinsed with Ringer solution. Imaging data were collected using a Till-Photonics imaging system (Till Photonics, Germany). We used a monochromatic light of 475 nm, dichroic of 500 nm, and emission LP of 515 nm. A sequence of 40 frames was acquired with a sampling rate of 4 Hz and total exposure time of 200 ms. Stimulation was set at frame 12 and lasted for 500 ms. The final size of the image was 320 × 240 pixels by binning 2 × 2 on chip. For the false color images, the relative calcium change of each frame was calculated as relative changes in fluorescence (Δ*F*/*F*) by MATLAB software. One hundred micrograms (10 μL of 10 μg/μL solutions) of Z9-16:Ald or Z11-16:Ald were used as stimuli, applied in random order. Paraffin oil was used as control.

### Immunocytochemistry

Brains were dissected in fresh Ringer’s solution (150 mM NaCl, 3 mM CaCl_2_, 3 mM KCl, 25 mM Sucrose, and 10 mM *N*-tris (hydroxymethyl)-methyl-2-amino-ethanesulfonic acid, pH 6.9). In order to visualize the AL glomeruli, immunostaining with anti-synapsin antibody SYNORF1 (Developmental Studies Hybridoma Bank, University of Iowa, Iowa City, IA, USA), was performed. The dissected brains were first fixed in a 4% paraformaldehyde solution in phosphate-buffered saline (PBS: 684 mM NaCl, 13 mM KCl, 50.7 mM Na_2_HPO_4_, 5 mM KH_2_PO_4_, pH 7.4) for 2 h at room temperature. After fixation, the brains were washed in PBS 4 × 15 min. To minimize non-specific staining, the brains were pre-incubated in 5% normal goat serum (Sigma, St. Louis, MO, USA) in PBS containing 0.5% Triton X-100 (PBSX; 0.1 M, pH 7.4) for 3 h before being incubated in the primary antibody, SYNORF1, at 1:100 in PBSX at 4°C for 5 days. Then the brains were rinsed in PBS 6 × 20 min, before being incubated in the secondary antibody, Cy2-conjugated anti-mouse (Invitrogen, Eugene, OR, USA; dilution 1:300 in PBSX), at 4°C for 3 days. The brains were finally rinsed 6 × 20 min in PBS, dehydrated with ascending ethanol series, and mounted in methylsalicylate.

### Confocal Image Acquisition and Digital 3D-Reconstruction

Serial optical images of the AL were obtained using a confocal laser scanning microscope (LSM 510, META Zeiss, Jena, Germany) with a 20× objective (Plan-Neofluar 20×/0.5l). A 488-nm line of an Argon laser was used to excite the Cy2. Confocal images were obtained with the resolution of 1024 × 1024 pixels at intervals of 3 μm.

To visualize the three-dimensional structure of the MGC, the confocal image stacks were subjected to reconstruction using the AMIRA software (AMIRA 5.3, Visage Imaging, Fürth, Germany). The structures were labeled as previously described using the segmentation editor, including the “brush” and “interpolate” tools (Berg et al., 2002; Skiri et al., 2005). The volumes of the individual glomeruli were measured by using the “tissue statistics” tool and imported to Excel for data processing.

### Data Analysis

Data of SSR were analyzed by the one-way ANOVA for analysis of variance, and the least significant difference (LSD) test was used for means multiple comparisons. The population ratios of sensilla types in the two parental species, hybrids and two backcrosses were compared using χ^2^ 2 × 2 test of independence with Yates’ continuity correction. χ^2^ test was used in comparison of segregation ratios. In calcium imaging, data were acquired by the software Till-vision (Till photonics) and further analyzed by software ImageJ (NIH, USA) and custom-made programs in MATLAB (The Math Works, Inc). One-way ANOVA and LSD were used to compare the response intensity of different glomeruli in the MGC of tested compounds.

## Results

### Inheritance of the Population Ratio of OSNs in Antennae Sensilla Tuned to Two Sex Pheromone Components

**FIGURE 1:**
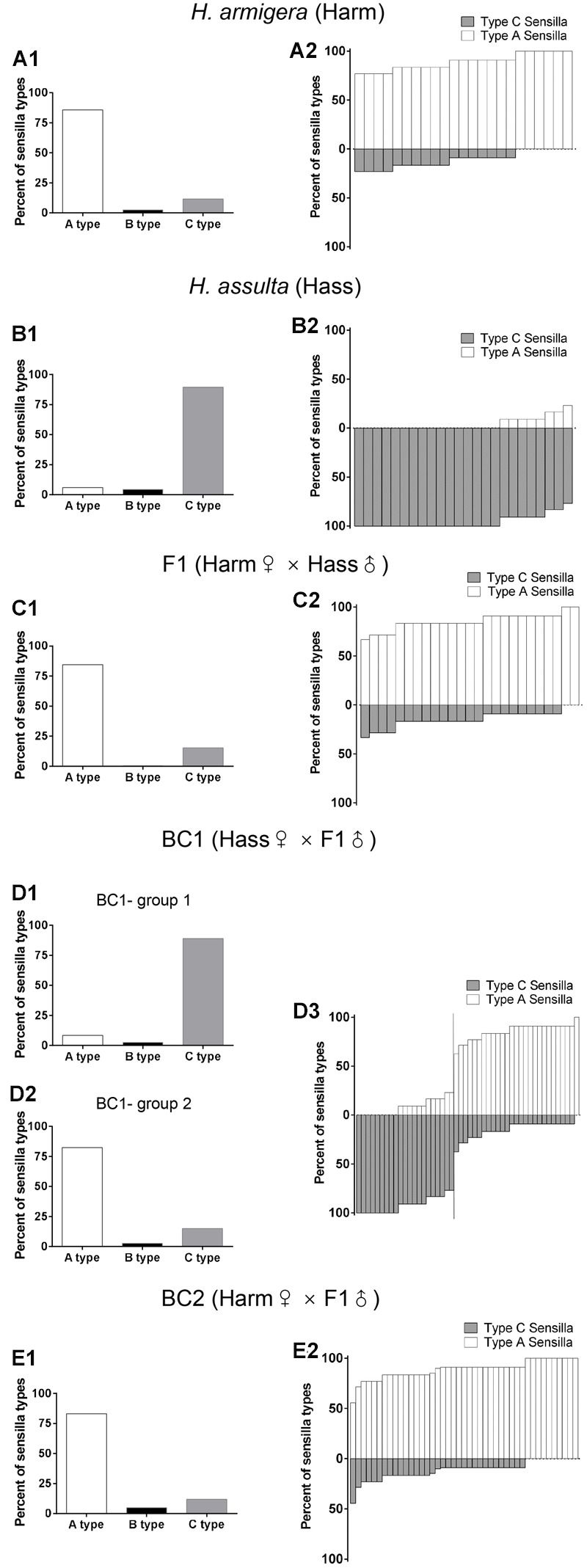
**Percent of A, B C sensilla types and percent of sensilla responsive to binary sex pheromone individually in male antennae of *H. armigera, H. assulta*, F1, and backcrosses.** The left columns **(A1,B1,C1,D1,D2,E1)** the percent of A, B and C sensilla types. The right columns **(A2,B2,C2,D3,E2)** the percent of A type sensilla responsive to Z11-16:Ald and C type sensilla responsive to Z9-16:Ald. In BC1 generation, individuals are divided into two groups according to their sensilla compositions. **(D1,D2)** the percent of A, B and C type sensilla in the two groups. A vertical line in **(D3)** separates the two groups.

For our study, we recorded the responses of 1934 male-specific trichoid sensilla from 163 individuals of *H. armigera, H. assulta*, F1, BC1, and BC2 (**Table 1**). Percentages of sensilla (A, B, and C type) in the two species, F1, BC1, and BC2, are reported in the left column of **Figure 1**. The percentages of sensilla containing OSNs responding to the sex pheromones Z11-16:Ald and Z9-16:Ald (housed in A type and C type sensilla, respectively) in each individual of two species, F1, BC1 and BC2 are presented in the right column of **Figure 1**.

**Table 1:** Ratios of A type sensila to C type sensilla in male antennae and ratios of areas responsive to Z11-16:Ald and Z9-16:Ald in male antennal lobes (ALs) of *H. armigera, H. assulta*, F1 (*H. armigera* × *H. assulta*), BC1 (*H. assulta* × F1), and BC2 (*H. armigera* × F1).

Cross	Number of individuals	Number of sensilla	Sensilla ratio^1^	Responsive area ratio^2^
*H. armigera*	23	268	7.42a	2.87a
*H. assulta*	24	267	0.07b	0.70b
F1	25	296	5.56a	3.06a
BC1- group 1	21	236	0.10b	0.47b
BC1-group 2	27	328	5.51a	2.20a
BC2	43	539	7.00a	2.30a
**Total**	163	1934		

*^1^Ratios in the same column having no letter in common are significantly different according to a χ^2^ 2 × 2 test of independence with Yates’ continuity correction (*P* < 0.05).*

*^2^Ratios in the same column having no letter in common are significantly different according to one-way ANOVA and LSD test (*P* < 0.05).*

In all *H. armigera* individuals A type sensilla were more abundant than C type, while the reverse was observed in *H. assulta*. All F1 and BC2 individuals presented the same pattern of sensilla as in *H. armigera*, but BC1 backcrosses could be segregated into two groups: group 1 containing 21 individuals with a majority of C type sensilla as in *H. assulta*, Group 2 containing 27 individuals with a majority of A type sensilla as in *H. armigera* (**Table 1**). The ratio between the two groups is close to 1 (χ^2^= 0.75, df = 1, *p* = 0.39).

### Changes of Response Spectra of Male-Specific Sensilla in F1 and Backcross Lines

**FIGURE 2:**
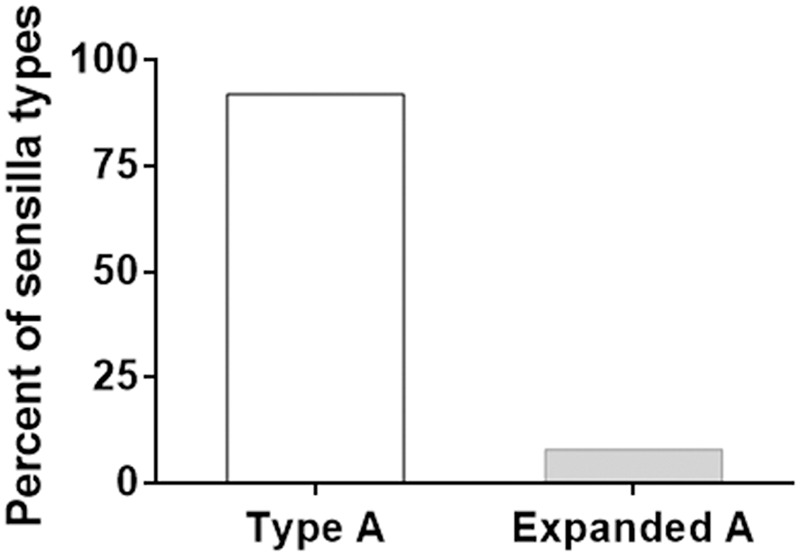
**A type and expanded A type sensilla in hybrid F1 male antennae.** The columns report percent of A type and “expanded A” type sensilla.

The A type sensilla in *H. armigera* and *H. assulta* only respond to Z11-16:Ald. In F1, 8% of A type sensilla, named “expanded A,” showed additional responses to Z11-16:OH and Z9-14:Ald (**Figure 2**; Supplementary Figure S2). No changes were found in the response spectra of A type sensilla in the two backcrosses. Compared to *H. armigera* and *H. assulta*, the response spectra of B type sensilla in F1, BC1, and BC2 did not show significant differences (**Table 2**).

**Table 2:** Responding patterns and numbers of subtypes of type B sensilla in *H. armigera, H. assulta*, F1, and backcrosses.

Subtype	Response compounds	*H. armigera*	*H. assulta*	F1	BC1	BC2
B1	Z9-14:Ald	-	6	-	11	10
Harm B2	Z9-14:Ald, Z11-16:OH	7	-	1	1	15
Hass B2	Z9-14:Ald, Z9-16:OH	-	6	-	3	2

**FIGURE 3:**
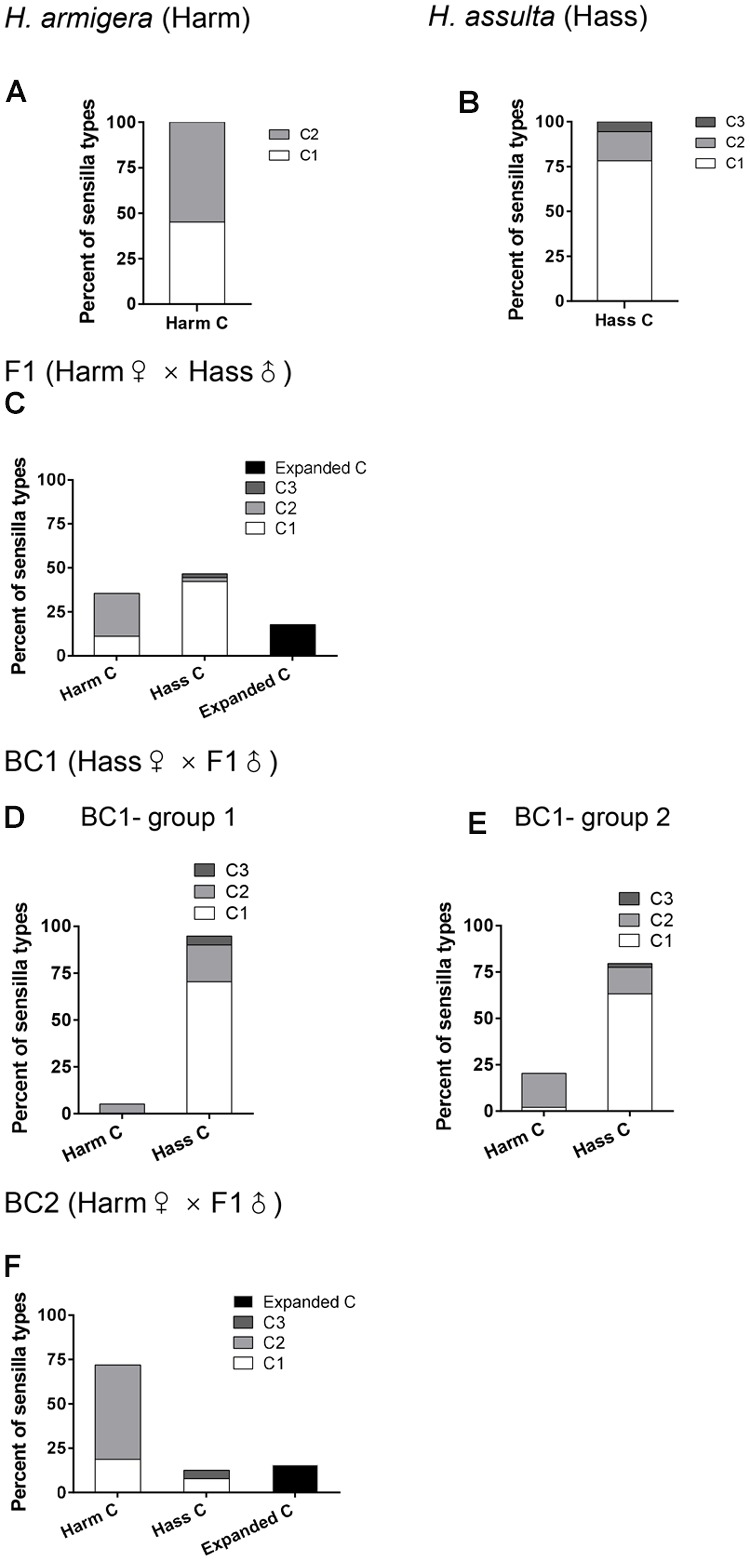
**Percent of subtypes of C type sensilla in male antennae of *H. armigera, H. assulta*, F1, and backcrosses.** The columns report percent for each subtype of C type sensilla. **(A–C,F)** the percent of subtypes of C type sensilla in *H. armigera, H. assulta*, F1, and BC2 respectively. **(D,E)** the percent of subtypes of C type sensilla in the two groups of BC1.

Responses of C type sensilla in F1 and backcrosses were much more complex (**Figure 3**). Two subtypes of *H. armigera* (Harm C1, Harm C2) and three subtypes of *H. assulta* (Hass C1, Hass C2, and Hass C3), as previously defined (Xu et al., 2016), were all found in F1 and backcrosses (**Figure 3**; Supplementary Figures S3 and S4). Supplementary Figures S5–S7 show the response patterns of the main subtypes of C type sensilla in F1, BC1, and BC2. It is worth noting that a new subtype of C sensilla named “expanded C” was found in F1 and BC2, with much broader response spectra (**Figures 3C,F**; Supplementary Figures S5 and S7). In F1, 35.6% of the C type sensilla responded as those in *H. armigera*, and 46.7% as those in *H. assulta*, while 17.8% belonged to the extended C type (**Figure 3C**; Supplementary Figure S5). In BC1, the majority of C type sensilla responded as those in the backcross parent, *H. assulta* (**Figures 3D,E**; Supplementary Figure S6). Similarly, in BC2, the majority of C type sensilla responded as those in the backcross parent, *H. armigera*, while the extended C type still accounted for 15.63% (**Figure 3F**; Supplementary Figure S7).

### Response Patterns of the Glomeruli in ALs to Two Sex Pheromone Components

**FIGURE 4:**
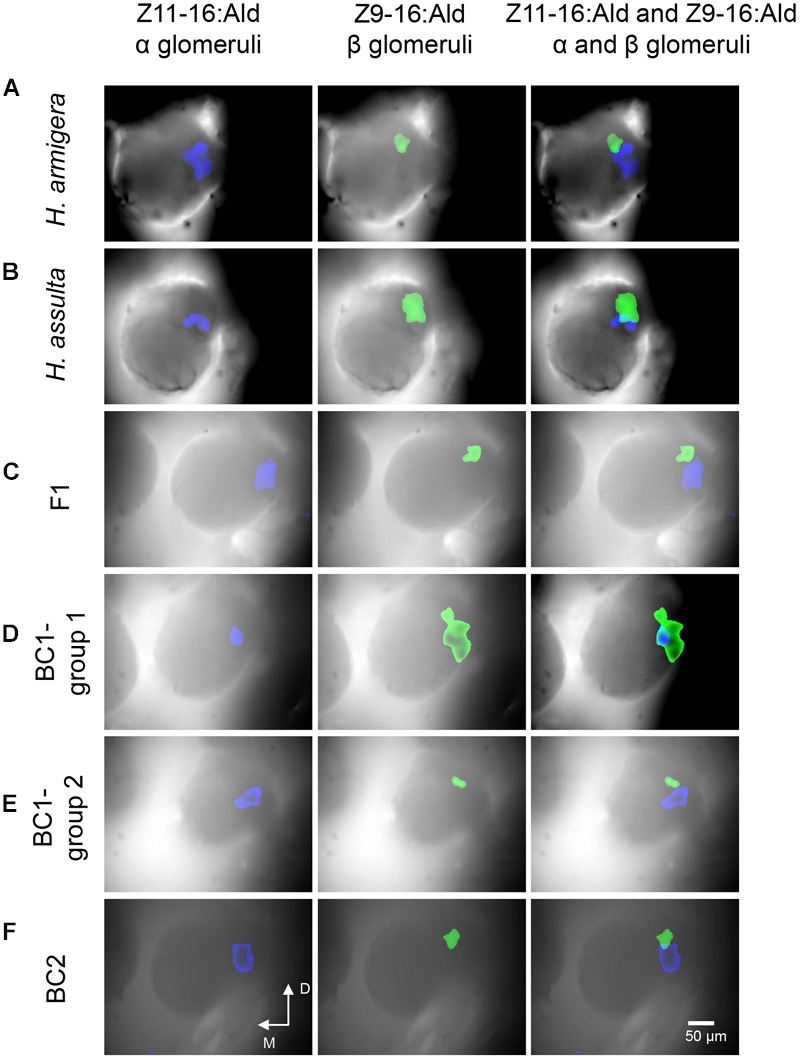
**Patterns of responses to Z11-16:Ald and Z9-16:Ald in the antennal lobe (AL) of *H. armigera, H. assulta*, F1, and backcrosses.** The antenna was stimulated with 100 μg of Z11-16:Ald and Z9-16:Ald. The glomerulus responsive to Z11-16:Ald is called α glomerulus (blue), the one responsive to Z9-16:Ald β glomerulus (green). **(A–F)** the corresponding activated patterns (exceeding 50% of maximum activity) superimposed on the gray-scale images of ALs (D, dorsal; M, medial).

Responses to sex pheromone components (Z11-16:Ald and Z9-16:Ald) in the first primary olfactory brain center, the ALs, were recorded using calcium imaging in seven males of *H. armigera* and seven of *H. assulta*, as well as in six hybrids and twelve of both BC1 and BC2. We defined the glomerulus responsive to Z11-16:Ald as α glomerulus and the one responsive to Z9-16:Ald as β glomerulus. In *H. armigera*, the α glomerulus was the largest unit, located close to the antennal nerve entrance of the MGC, whereas the β glomerulus was smaller and located at the posterior dorsomedial region (**Figure 4A**). In *H. assulta*, on the other hand, the β glomerulus was largest and positioned close to the antennal nerve entrance, while the small α glomerulus was located at the ventral region (**Figure 4B**).

The topography in hybrids and BC2 males was similar to that of *H. armigera* (**Figures 4C,F**). The ratios of the areas activated by Z11-16:Ald (α glomerulus) and by Z9-16:Ald (β glomerulus) in F1 and BC2 were also similar to those of *H. armigera* (**Table 1**). However, the situation was quite different among BC1 males. Six individuals (BC1- group 1) showed a topography similar to that of *H. assulta* (**Figures 4B,D**) with a similar ratio between the areas of α and β glomeruli (**Table 1**). On the contrary, the other six BC1 males (BC1- group 2) were similar to *H. armigera* (**Figures 4A,E**) both in responsive topographic structures of MGC and ratios of responsive areas (**Table 1**).

### 3D Structure of MGC in F1 and Parents

**FIGURE 5:**
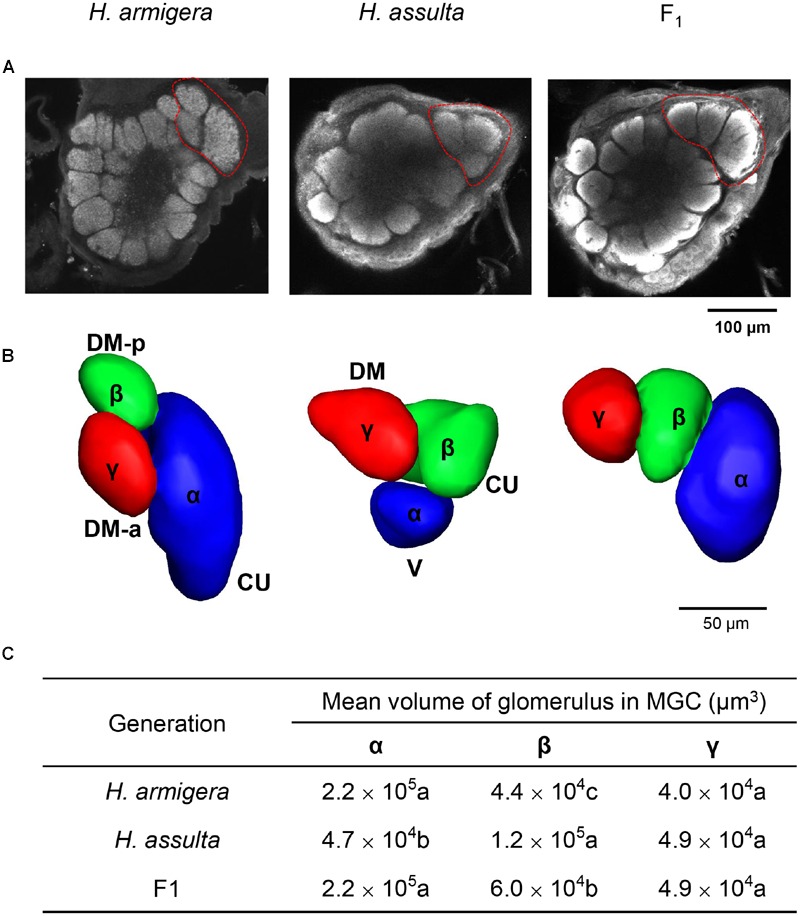
**3D structure of AL and reconstruction of macroglomerular complex (MGC) areas of *H. armigera, H. assulta*, and hybrid F1. (A)** Confocal image of the 3D structure of AL glomeruli in frontal view. The MGC areas are encircled within red dotted line; **(B)** 3D reconstruction of MGC areas based on the confocal stacks of the brain shown in **(A)**. We renamed the three glomeruli of the MGC areas. Besides α and β glomeruli (**Figure 4**), there is a third γ glomerulus; the α and β glomerulus in F1 were identified based on the results of calcium imaging (**Figure 4**). **(C)** A summary of the volumes of three glomeruli is given. Volumes in the same column having no letter in common are significantly different. (Cu, Cumulus; DM-p, posterior dorsomedial unit; DM-a, anterior dorsomedial unit; DM, dorsomedial unit).

The MGC areas of the two parents and F1 hybrids, all consisting of three units are shown in **Figure 5**. Besides α and β glomeruli, a third glomerulus was found and named γ glomerulus. The identity of each glomerulus in F1 AL has been assigned in the calcium imaging map (**Figure 4**). The topography of F1 was similar to but not identical with that of *H. armigera*. The volumes of three units in MGC of F1 are similar (**Figure 5C**): the α and γ glomerulus in F1 did not show significant differences in size with those in *H. armigera*, while the β glomerulus was larger.

## Discussion

Two sister species, *H. armigera* and *H. assulta* share the same pheromone components in reverse ratios. How the two species recognize their sex pheromone blends and achieve behavioral isolation is an issue that has been intensively investigated (Wang et al., 2005; Wu et al., 2013; Xu et al., 2016). The two species could be successfully hybridized in the laboratory (Wang and Dong, 2001; Wang et al., 2004; Zhao et al., 2006) and the fertile hybrids provide us with an unique opportunity to study the genetic basis of sex pheromone detection. In this study, we used SSR, calcium imaging, immunocytochemistry and digital 3D-reconstruction to comparatively study the pheromone detection in the two sister species, their hybrids and backcrosses. We found that the inheritance of the population ratio between the two types of sensilla tuned to two pheromone components is controlled by a major gene, and the allele of *H. armigera* is dominant. The sensory system of F1 and BC2 for sex pheromones are similar to that of *H. armigera*, but the response profile of some sensilla in male antennae of F1 and BC2 is broader than that in *H. armigera*. These results imply that introgression might occur from *H. assulta* into *H. armigera* through repeated back-crossing when hybridization of the two species occurs in the field.

### Inheritance of the Population Ratio of Sensilla Responsive to Sex Pheromone and the Proportion of Responsive Areas in the AL

In moth species, the majority of OSNs are usually tuned to the most abundant pheromone component (Baker et al., 2012). In *H. armigera* and *H. assulta*, OSNs tuned to the binary sex pheromone blends Z11-16:Ald and Z9-16:Ald are classified into type A and C sensilla (Berg and Mustaparta, 1995; Wu et al., 2013). Also, the population ratios of A type to C type sensilla in these two species are positively correlated with their pheromone components proportions (Wu et al., 2013). Consequently, the glomerulus innervated by OSNs responsive to Z11-16: Ald is larger in *H. armigera* than in *H. assulta* (Wu et al., 2013).

Electrophysiological recordings in hybrid males of two strains, E and Z, of *O. nubilalis* revealed that the ratios of spike amplitude between OSNs responding to sex pheromones exhibits E-strain dominance. In a similar study on *H. virescens* and *H. subflexa*, hybrid male OSNs appeared to be more similar to *H. subflexa* than *H. virescens* (Baker et al., 2006; Olsson et al., 2010). In this study, the ratios of OSNs responding to Z11-16:Ald and Z9-16: Ald between A type and C type sensilla of RS hybrids and BC2 males were consistent with that in their parental species *H. armigera*, while in BC1, separation occurred and half of them were similar to *H. armigera*, the other half similar to *H. assulta*. The same situation was also observed in the ALs. Topology and relative sizes of the related glomeruli of MGC in hybrid RS and BC2 were consistent with the parental species *H. armigera*, while in BC1, half of the individuals were similar to *H. armigera*, the other half to *H. assulta*. 3D-structure and volumes of glomeruli in the MGC of F1 was intermediate between the two species, with α glomerulus similar in size to that of *H. armigera*. These results clearly show that the population ratio of two pheromone-sensitive OSNs is controlled by a major gene, and the allele of *H. armigera* is dominant, which is consistent with the inheritance pattern of sex pheromone composition identified in the two species. The female hybrids from female *H. assulta* and male *H. armigera* produce pheromone components in a ratio similar to that of *H. armigera* (Wang et al., 2005).

### Response Spectra of the Antennal Sensilla in F1 and Backcrosses

The inheritance of the response spectra of C type sensilla in the two species is quite complex. In F1 and in the backcrosses, C type sensilla of the two species coexist on the same antenna. New sensilla subtypes with expanded response profiles were identified in the hybrids and the backcrosses. Eight percentage of A type sensilla in F1 showed broader spectra of response. A similar change in A type sensilla has been previously reported in the hybrids of *H. virescens* and *H. subflexa* (Baker et al., 2006). Sensilla of the new expanded C subtype were detected in F1 and BC2. These sensilla responded to five compounds, while the C type sensilla of *H. assulta* and *H. armigera* just responded to three or four of them. The change in the response spectra of male-specific sensilla in hybrids and backcrosses could derive from genetic recombination of the two sister species (Baker et al., 2006; Koutroumpa et al., 2014). Different pheromone receptors co-expressed or co-localized in two sensory neurons in C type sensilla might lead to variations in the response spectra of sensilla and thus produce different sensilla subtypes (Berg and Mustaparta, 1995; Berg et al., 2005; Wu et al., 2013; Xu et al., 2016).

Emergence of a new subtype of sensilla in hybrids and backcrosses suggests that the olfactory response spectra of sensilla possess a great genetic flexibility, which might enhance adaptation of the hybrids to the environment and play an important role in species evolution. Female moths experience lower pressure and thus can produce a larger variety of pheromone components and their ratios (Phelan, 1992; Roelofs et al., 2002), while the male broader sensory range could follow the female variations, thus adapting to the new pheromone components or ratios (Baker, 2002).

### Fates of the Hybrids and Gene Flow between *H. armigera* and *H. assulta*

Hybridization is considered to be an alternative way of speciation although it is quite rare in nature (Rieseberg, 1997; Buerkle et al., 2000). However, interspecific hybrids were found to occur occasionally from two closely related butterflies, *Heliconius cydno* and *Heliconius melpomene* which share the same habitat; the hybrid *Heliconius heurippa* was recognized as a new species due to its unique wing pattern (Mavarez et al., 2006). In the cross experiments of *H. armigera* and *H. assulta* carried out in the last 2 years, the hybrids from female *H. armigera* and male *H. assulta* (RS hybrids) were obtained with around 4–10% success rate, while the reciprocal cross hybrids from male *H. armigera* and female *H. assulta* (SR hybrids) were not obtained after more than 30 attempts. Therefore, RS hybrids are much easier to produce than SR hybrids. RS hybrid males produced in the field are most likely attracted to *H. armigera* females because of their response to a mixture of Z11-16:Ald and Z9-16:Ald similar to that of male *H. armigera*. The resulting backcross (BC2) presents a very high success rate (93%, unpublished data), and thus the BC2 line is the inevitable offspring of RS hybrids. Because BC2 individuals are much more similar to *H. armigera* in their male olfactory sensory system (this study) and female sex pheromone synthesis (Wang et al., 2008), they would stand a good chance to backcross again with *H. armigera*. After a number of successive backcrosses, the offspring would contain some specific traits of *H. assulta* in the genetic background of *H. armigera.* New types of sensilla with broader response profile may be selected and males could become further adaptive to variations in the female sex pheromone. Introgression might occur from *H. assulta* into *H. armigera* through repeated back-crossing, and eventually would increase the fitness of *H. armigera*.

## Conclusion

We demonstrate that the population ratio of two OSNs in A and C type sensilla responding to the two main pheromone components of *H. armigera* and *H. assulta*, is controlled by a major gene, and the allele of *H. armigera* is dominant. New subtypes of sensilla with broader response spectra were found in interspecific crossing and backcrossing offspring. We predict that in case hybridization of female *H. armigera* and male *H. assulta* occurs in the field, male hybrids would readily backcross with female *H. armigera* because of similarities in their pheromone sensory system with that of male *H. armigera.* If true, this would imply impossibility of hybrid speciation through hybridization of the two species, but introgression may happen from *H. assulta* into *H. armigera* through repeated backcrossing. The great success of *H. armigera* in the Old World may be related to the specific genetic mechanisms of pheromone communication in this group of insect species.

## Author Contributions

C-ZW, MX, J-FD, and HW designed research, MX, HW, and X-CZ performed research, J-FD and L-QH contributed materials and technical support, C-ZW, MX, HW, and X-CZ analyzed data, C-ZW and MX wrote the paper.

## Conflict of Interest Statement

The authors declare that the research was conducted in the absence of any commercial or financial relationships that could be construed as a potential conflict of interest.
