# Supplementary material for: The Inheritance of the Pheromone Sensory System in Two Helicoverpa Species: Dominance of H. armigera and Possible Introgression from H. assulta
Source: Front Cell Neurosci. 2017 Jan 10;10:302. doi: 10.3389/fncel.2016.00302 (PMC5222888; doi:10.3389/fncel.2016.00302)

*Supplementary Material*

# The inheritance of the pheromone sensory system in two *Helicoverpa* species: dominance of *H. armigera* and possible introgression from *H. assulta*

**Meng Xu*, Jun-Feng Dong*, Han Wu, Xin-Cheng Zhao, Ling-Qiao Huang, Chen-Zhu Wang**

*** Correspondence:** Chen-Zhu Wang: czwang@ioz.ac.cn

*These authors contributed equally to this study.

**Supplementary Figures**

**Supplementary Figure 1. The morphology of male antennae in *Helicoverpa* species and the antennal region for single sensillum recording in this study.** A: a whole antenna of male *H. assulta*. Red line indicates the range of the flagellar segments from the basal $20^{th}$ to $60^{th}$ of the antenna. Scale bar: 1 mm. B: Male-specific trichoid sensilla on the antenna of *H. assulta*. Arrow indicates trichoid sensilla. Scale bar: 100 µm. The same for male *H. armigera*.

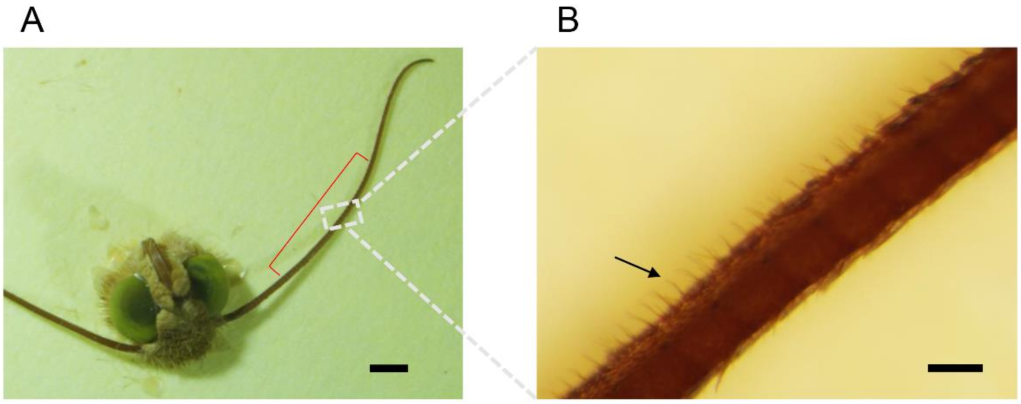

**Supplementary Figure 2. Response patterns of the associated olfactory receptor neurons housed in A type and expanded A type sensilla in male antennae of F1.** For each subtype of sensilla, the left columns **(A, C)** show examples of the electrophysiological recordings. Black bars at the top of each column show the duration of the stimuli. The right columns **(B, D)** report the spike frequencies (Mean ± SEM) which were calculated by counting the number of spikes during the first 200 ms of the response.

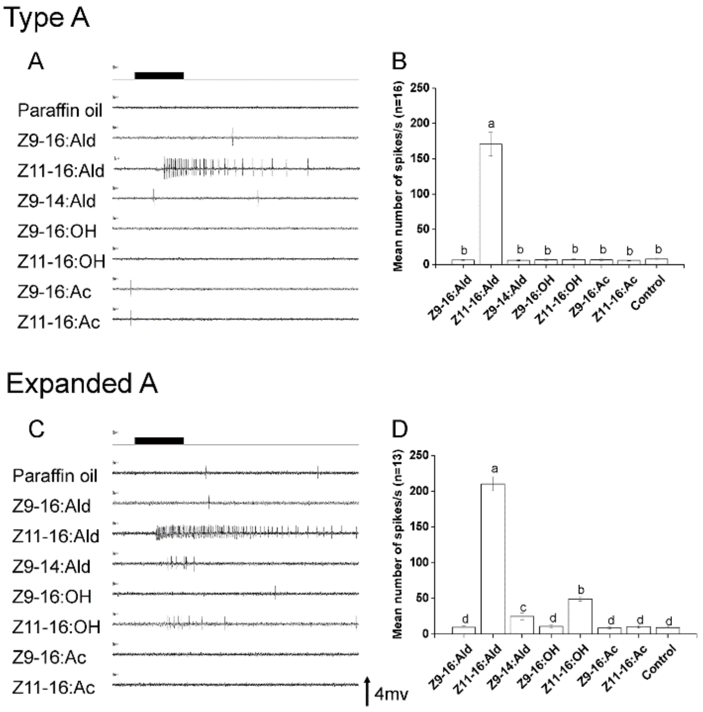

**Supplementary Figure 3. Response patterns of the associated olfactory receptor neurons housed in two subtypes of C type sensilla in male antennae of *H. armigera*.** For each subtype of sensilla, the left columns **(A, C)** show examples of the electrophysiological recordings. Black bars at the top of each column show the duration of the stimuli. The right columns **(B, D)** report the spike frequencies (Mean ± SEM) which were calculated by counting the total number of spikes during the first 200 ms of the response, because this type sensilla contain two OSNs with similar spike amplitudes (Wu et al., 2015). The total number of spikes from two co-localized neurons was counted. Columns with the same letters are not significantly different at $P < 0.05$. Paraffin oil was used as control.

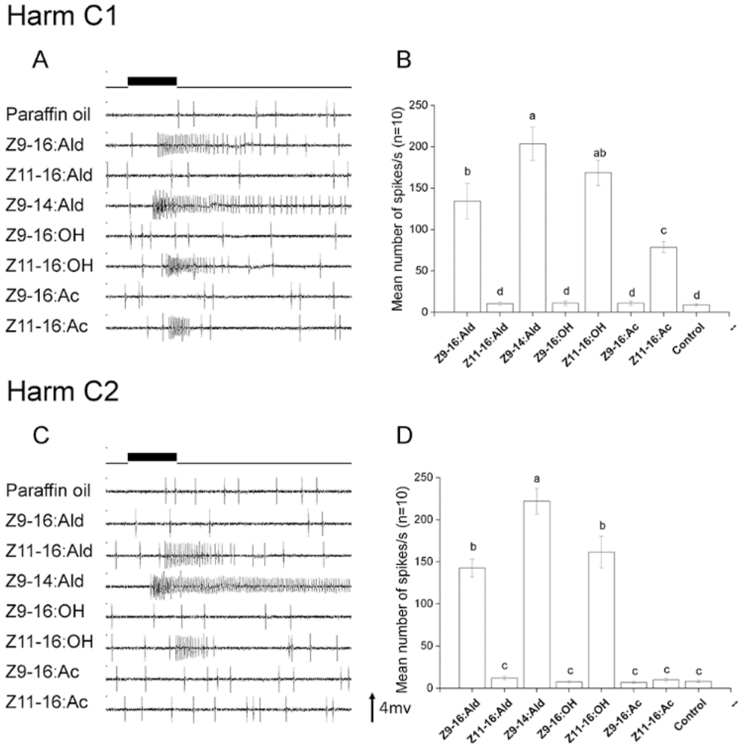

**Supplementary Figure 4. Response patterns of the associated olfactory receptor neurons housed in three subtypes of C type sensilla in male antennae of *H. assulta*.** For each subtype of sensilla, the left columns **(A, C, E)** show examples of the electrophysiological recordings. Black bars at the top of each column show the duration of the stimuli. The right columns **(B, D, F)** report the spike frequencies (Mean ± SEM) which were calculated by counting the number of spikes during the first 200 ms of the response. Columns with the same letters are not significantly different at $P < 0.05$. Paraffin oil was used as control.

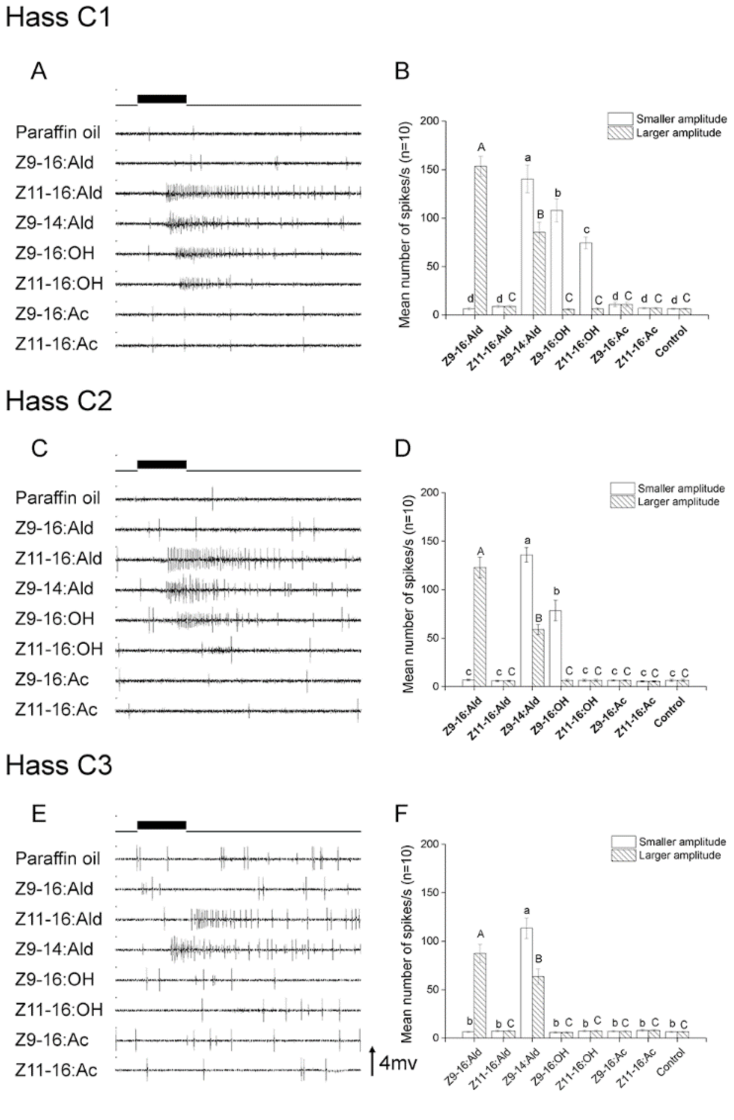

**Supplementary Figure 5. Response patterns of the associated olfactory receptor neurons housed in main subtypes of C type sensilla in male antennae of hybrids F1.** For each subtype of sensilla, the left columns **(A, C, E, G)** show examples of the electrophysiological recordings. Black bars at the top of each column show the duration of the stimuli. The right columns **(B, D, F, H)** report the spike frequencies (Mean ± SEM) which were calculated by counting the number of spikes during the first 200 ms of the response. The total number of spikes from two co-localized neurons was counted. Columns with the same letters are not significantly different at $P < 0.05$. Paraffin oil was used as control.

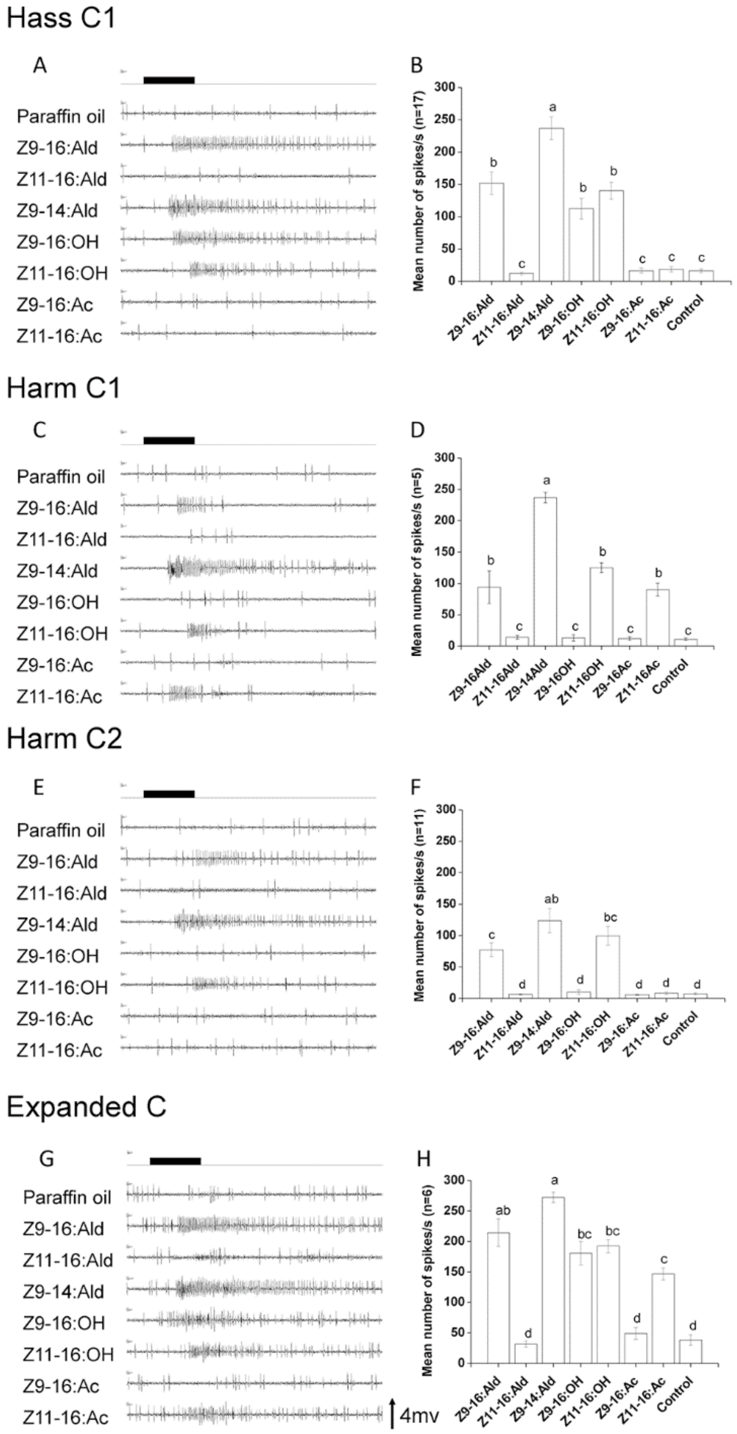

**Supplementary Figure 6. Response patterns of the associated olfactory receptor neurons housed in main subtypes of C type sensilla in male antennae of BC1.** For each subtype of sensilla, the left columns **(A, C, E)** show examples of the electrophysiological recordings. Black bars at the top of each column show the duration of the stimuli. The right columns **(B, D, F)** report the spike frequencies (Mean ± SEM) which were calculated by counting the number of spikes during the first 200 ms of the response. The total number of spikes from two co-localized neurons was counted. Columns with the same letters are not significantly different at $P < 0.05$. Paraffin oil was used as control.

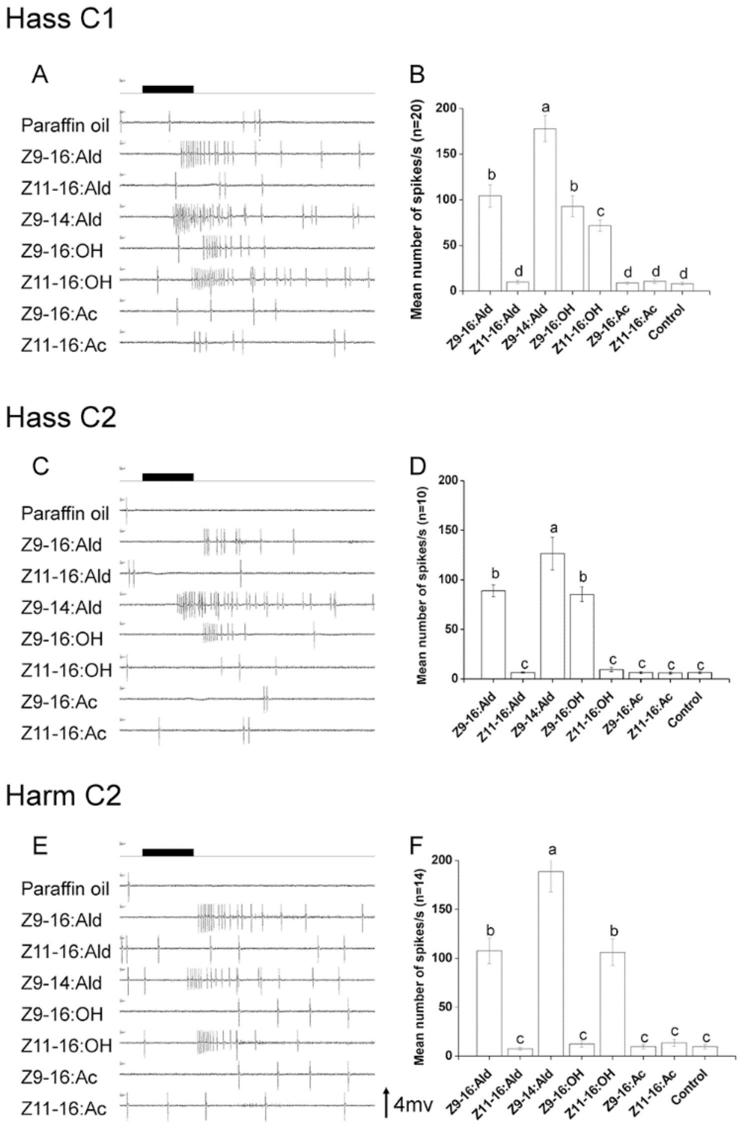

**Supplementary Figure 7. Response patterns of the associated olfactory receptor neurons housed in main subtypes of C type sensilla in male antennae of BC2.** For each subtype of sensilla, the left columns **(A, C, E)** show examples of the electrophysiological recordings. Black bars at the top of each column show the duration of the stimuli. The right columns **(B, D, F)** report the spike frequencies (Mean ± SEM) which were calculated by counting the number of spikes during the first 200 ms of the response. The total number of spikes from two co-localized neurons was counted. Columns with the same letters are not significantly different at $P < 0.05$. Paraffin oil was used as control.

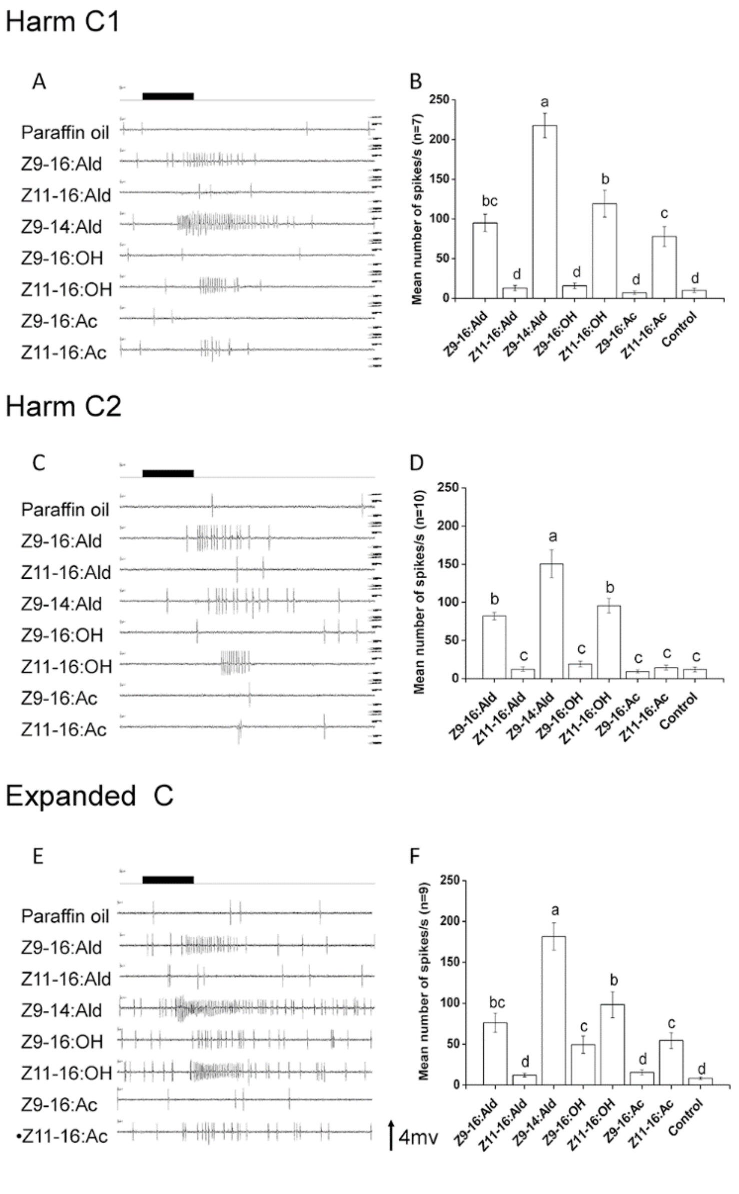

Supplement: Supplementary file 1 [file Presentation_1.pdf]
